# Complete plastome sequence of *Cymbidium tortisepalum* var. *longibracteatum* (Y.S.Wu & S.C.Chen) S.C.Chen & Z.J.Liu (Orchidaceae): an endangered (EN) plant species in Southwest China

**DOI:** 10.1080/23802359.2020.1810157

**Published:** 2020-08-17

**Authors:** Da-Juan Chen, Hong-Xin Wang, Zhi-Xin Zhu, Hua-Feng Wang

**Affiliations:** Hainan Key Laboratory for Sustainable Utilization of Tropical Bioresources, College of Tropical Crops, Hainan University, Haikou, China

**Keywords:** *Cymbidium tortisepalum* var. *longibracteatum*, plastome, phylogeny, genome structure, Orchidaceae

## Abstract

*Cymbidium tortisepalum* (Orchidaceae) has been ranked as an endangered (EN) herb species in China. In this study, we report and characterize the complete plastid genome sequence of *C. tortisepalum* var. *longibracteatum* in order to provide genomic resources helpful for promoting its conservation and garden utilization. The complete plastome is 150,198 bp in length and contains the typical quadripartite structure of angiosperm, including two Inverted Repeat (IRs) regions of 25,682 bp, a Large Single-Copy (LSC) region of 85,035 bp and a Small Single-Copy (SSC) region of 13,799 bp. The plastome contains 111 genes, consisting of 77 unique protein-coding genes, 30 unique tRNA gene and 4 unique rRNA genes. The overall A/T content in the plastome of *C. tortisepalum* var. *longibracteatum* is 62.90%. The complete plastome sequence of *C. tortisepalum* var. *longibracteatum* will provide a useful resource for the conservation and garden utilization of this species as well as for the phylogenetic studies of Orchidaceae.

## Introduction

*Cymbidium tortisepalum* var. *longibracteatum* (Y.S.Wu & S.C.Chen) S.C.Chen & Z.J.Liu is a herb species belonging to Orchidaceae. Leaves are stiff and suberect, 50–70 cm long and 1.2–1.5 cm wide. *C. tortisepalum* var. *longibracteatum* is native to Guizhou, Sichuan and Yunnan of China and it grows in rocky and scrubby slopes with altitude from 1000–2000 m (Liu et al. 2009). It has been ranked as an endangered (EN) species in China (Qin et al. 2017). Consequently, the genetic and genomic information is urgently needed to promote its systematics research and the development of conservation value of *C. tortisepalum* var. *longibracteatum.* In this study, the complete plastome of *C. tortisepalum* var. *longibracteatum* (GenBank accession number: MT747170) was reported and characterized. This is the first report of a complete plastome for *C. tortisepalum* var. *longibracteatum.*

In this study, *C. tortisepalum* var. *longibracteatum* was sampled from the greenhouse within Hainan University campus, Haikou, Hainan, China (110.33°E, 20.06°N). A voucher specimen (Wang et al. B260) was deposited in the Herbarium of the Institute of Tropical Agriculture and Forestry (HUTB), Hainan University, Haikou, China.

The experiment procedure is as reported in Zhu et al. (2018). Around six Gb clean data were assembled against the plastome of *Cymbidium tortisepalum* (NC_021431.1) (Yang et al. 2013) using MITObim v1.8 (Hahn et al. 2013). The plastome was annotated using Geneious R8.0.2 (Biomatters Ltd., Auckland, New Zealand) against the plastome of *C. tortisepalum* (NC_021431.1).

The plastome of *C. tortisepalum* var. *longibracteatum* is found to possess a total length 150,198 bp with the typical quadripartite structure of angiosperms, contains two Inverted Repeats (IRs) of 25,682 bp, a Large Single-Copy (LSC) region of 85,035 bp and a Small Single-Copy (SSC) region of 13,799 bp. The plastome contains 111 genes, consisting of 77 unique protein-coding genes, 30 unique tRNA genes and 4 unique rRNA genes. The overall A/T content in the plastome of *C. tortisepalum* var. *longibracteatum* is 62.90%, which the corresponding value of the LSC, SSC and IR region were 65.50%, 70.30% and 56.50%, respectively.

**Figure 1.**
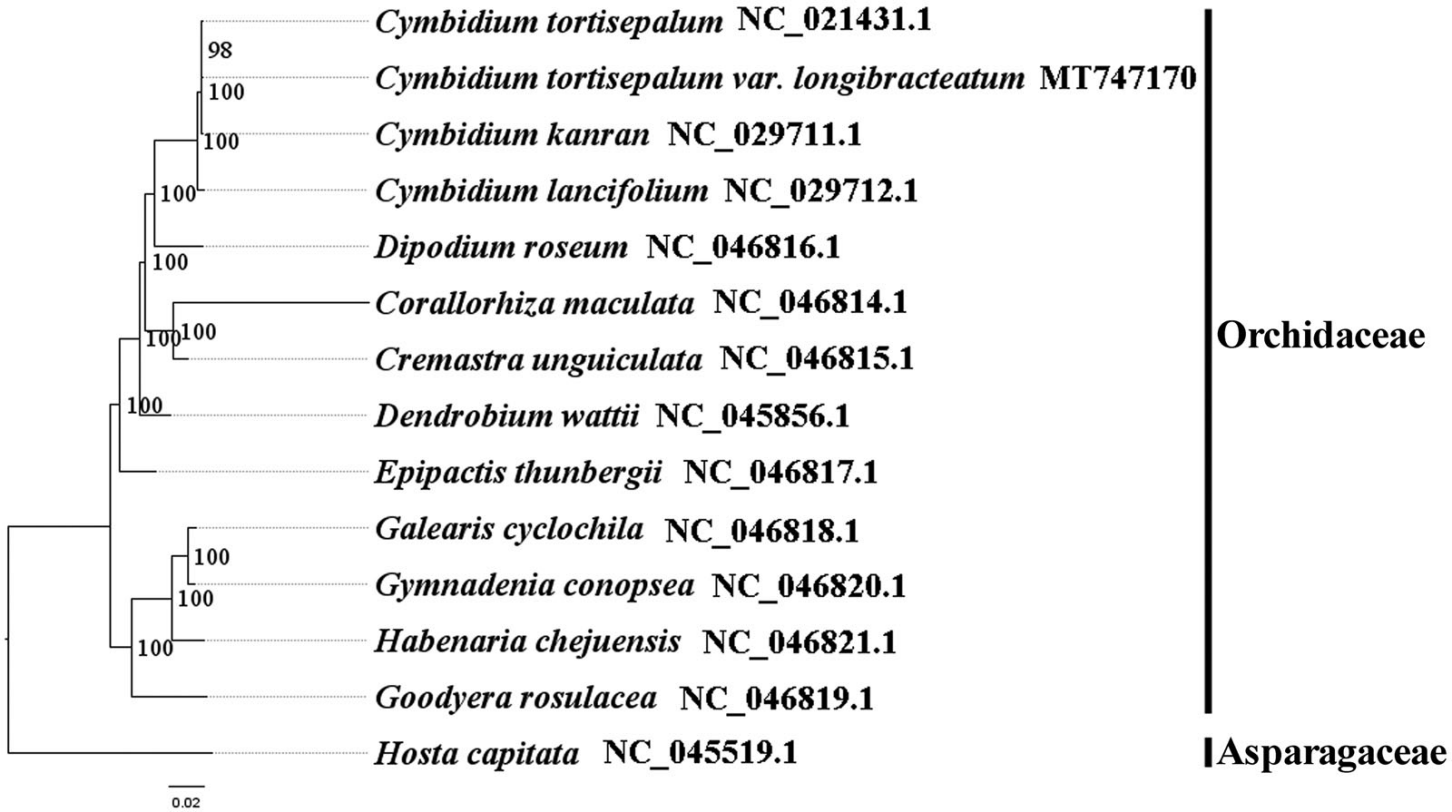
The best ML phylogeny recovered from 14 complete plastome sequences by RAxML. Accession numbers: *Cymbidium tortisepalum* var. *longibracteatum* MT747170, *Cymbidium tortisepalum* NC_021431.1, *Cymbidium kanran* NC_029711.1, *Cymbidium lancifolium* NC_029712.1, *Dipodium roseum* NC_046816.1, *Corallorhiza maculata* NC_046814.1, *Cremastra unguiculata* NC_046815.1, *Dendrobium wattii* NC_045856.1, *Epipactis thunbergii* NC_046817.1, *Galearis cyclochila* NC_046818.1, *Gymnadenia conopsea* NC_046820.1. *Habenaria chejuensis* NC_046821.1, *Goodyera rosulacea* NC_046819.1. Outgroups: *Hosta capitata* NC_045519.1.

We used RAxML (Stamatakis 2006) with 1000 bootstraps under the GTRGAMMAI substitution model to reconstruct a maximum likelihood (ML) phylogeny of 12 published complete plastomes of Orchidaceae, using *Hosta capitata* (Asparagaceae) as an outgroup. The phylogenetic analysis indicated that *C. tortisepalum* var. *longibracteatum* is close to *C. tortisepalum* within Orchidaceae in this study (Figure 1). Most nodes in the plastome ML tree were strongly supported. The complete plastome sequence of *C. tortisepalum* var. *longibracteatum* will provide a useful resource for the conservation genetics of this species as well as for the phylogenetic studies of Orchidaceae.

## Data Availability

The data that support the findings of this study are openly available in GenBank of NCBI at http://www.ncbi.nlm.nih.gov, reference number MT747170.
